# Numerical Analysis of Protection Method of Metallic Sub-Wavelength Concentric Arrays for Radially Polarized Light Selection and Its Applications

**DOI:** 10.3390/s21134480

**Published:** 2021-06-30

**Authors:** Hyuntai Kim

**Affiliations:** Electrical and Electronic Convergence Department, Hongik University, Sejong 30016, Korea; hyuntai@hongik.ac.kr

**Keywords:** radially polarized light, nano-photonics, binary plate, protective layer

## Abstract

Radially polarized light has various advantages on sensing, thanks for its symmetric field distribution. To select radial component, metallic sub-wavelength concentric arrays are widely used. To increase the stability of the metallic nanostructure from mechanical or chemical hazards, a method to apply an additional protective layer has been proposed. The structure was numerically calculated, and optimized structure showed ~97.4% of transmittance for radially polarized component with ~20 dB of polarization extinction ratio compared to the azimuthally polarized component. This result is a 22% increase compared to the case without the protective layer. In addition, the utility the protective layer applied to metallic sub-wavelength concentric arrays is also discussed. The structure has been applied to a binary, concentric optical plate, and showed the same function with radially polarized input, but prohibited azimuthally polarized input. The proposed structure is expected to be applied on numerous centrosymmetric flat optical components.

## 1. Introduction

Radially polarized (RP) light has numerous applications in the sensing field thanks to its relatively small spot size and radially symmetric electric field [1,2,3,4,5]. However, unlike general polarization states, RP has a different polarization direction depending on spatial position, so it is challenging to make an RP state with common methods such as using a general waveplate or linear polarizer. Various methods have been introduced for detection or decomposition of RP modes [6,7,8]. Metallic sub-wavelength concentric ring arrays (MSCRA) are one of the conventional structures to select such RP lights [9,10,11]. MSCRA has a structure and principle similar to that of a linear polarizer, but it is a centrosymmetric structure. MSCRA has advantages in that eases of manufacture, and having a high polarization contrast, i.e., polarization extinction ratio (PER) [12]. In addition, MSCRA is applicable to other binary optical components which have axial symmetry, and allows only RP component light to be transmitted, while retaining its desired functions [10,13].

Most of the cases, MSCRA is attached at the input or output end of the waveguide, which allows only RP component to be entered or exited from the waveguide. However, the MSCRA structure has various maintenance issues such as oxidation or mechanical damage [14].

Therefore, a method to protect the MSCRA layer is suggested. By adding an additional protective dielectric layer, the metallic nano-structured layer is secure from external force or chemical damages. In addition, it will be possible to optimize the transmittance by selecting the material of the protective layer and adjusting the thickness [15,16]. In particular, when the refractive index of the substrate material is high, choosing a material that has a lower refractive index than that of the substrate material, rather than making a protective layer with the same material, could increase the efficiency by reducing the reflectance.

In this paper, the characteristic of the protective layer is calculated via numerical simulations. Both directions, from waveguide to free-space and from free-space to waveguide are considered. The efficiency of transmission is optimized considering proper PER level. In particular, this paper analyzed the selection of protective layer materials and the control of its thickness to secure low efficiency when nanostructures are applied to the surface of high refractive index. In addition, optical component design applying the protected MSCRA structure is also proposed.

## 2. Materials and Methods

**Figure 1:**
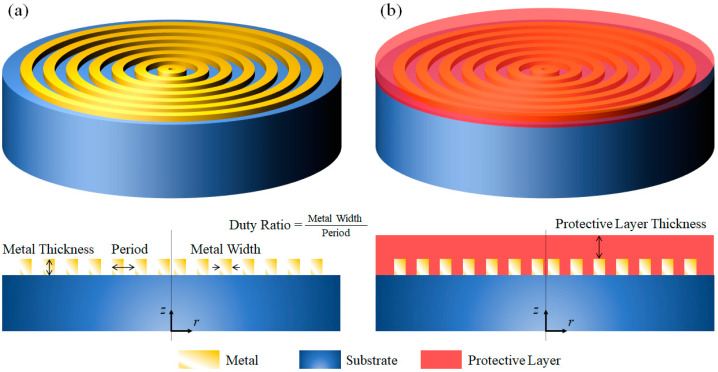
(**a**) Configuration of the structure of MSCRA without protective layer. (**b**) Configuration of the protective layer applied MSCRA.

MSCRA structure generally prevents azimuthally polarized (AP) light component but allows RP light component. Note that RP light refers to light in which the electric field at all positions is directed toward or off the center, i.e., radial direction. AP light refers to light in which the electric field at all positions revolves in azimuthal (concentric) direction or revolves/orbits around the center [6,10]. The concentric ring has similar principles to the nano-slit array polarizer, but with axial symmetry. The physical origin of its operation—transmitting RP component while reflecting AP component—can be explained by the nature of electron movement [17], by plasmonic generation [18], or also by effective medium theory [19]. The MSCRA structure also shares similar maintenance issues as the wire grid polarizer. To enhance the stability of the metallic layer, an additional shielding layer is proposed. The structure is shown in Figure 1. Figure 1a shows the general MSCRA structure. However, it is clear that the nano-structured metallic region is exposed at the free-space, which has potential risks of oxidization, dust issue, or physical damage. Therefore, an additional protective layer such as shown in Figure 1b will increase its stability. The structure also depicts parameters such as metal thickness, period, duty ratio, and protective layer thickness which would be referred to in further discussion.

First, the transmission from dielectric region to free-space and free-space to dielectric are both optimized. The dielectric material is assumed to be silicon, which is a typical material for waveguide. In addition, the silicon waveguide has high surface reflection loss, and the structure is also designed to minimize the reflection loss. The protected structure is similar to a 4-layer structure of substrate-metallic region protective layer-free-space. When each refractive index gradually increases, antireflection design becomes more effective. Therefore, the protective layer can also be made of silicon [20], but if so, the reflection from the surface cannot be easily minimized. Thus, the metal selected is gold [21], and silica has been chosen as the protective layer [22], which has a refractive index between air and silicon. Finite element method simulations via COMSOL Multiphysics have been performed. The simulations were performed based on axial symmetry mode. The outer region of the simulation was assumed to be a perfect matching layer [23]. The resolution of the simulation was < λ/6*n* for common regions where n is the refractive index of the material, < λ /25 for near the focal position, and < λ /60 near the metallic regions. Note that the lower duty ratio, which is the ratio of metallic region, results in higher transmission because of low ohmic loss. However, if the duty ratio is lower than 0.2, the AP component can also transmit the metallic layer. Therefore, a duty ratio of 0.25 has been assumed for further numerical calculations, which is shown in ~20 dB of PER. Note that 20 dB of PER represents <1% of unwanted polarization component. Numerous research regarded 20 dB of value as a reasonable value [24,25].

In addition, the application method of the proposed protected MSCRA structure is considered. The MSCRA structure can be applied to other concentric binary optical components and can perform regularly and with additionally transmitting only the RP component. In this paper, a dual on-axis RP focusing lens has been designed with applying the protective MSCRA layer. The lens was designed based on virtual point method and the field after the protective layer applied MSCRA lens is calculated via numerical simulations based on finite element method.

## 3. Results

### 3.1. Protective MSCRA Layer Optimization

First, using COMSOL, the transmittance was optimized under the condition that the PER was maintained at ~20 dB level. The transmission when RP Bessel beam input and AP Bessel beam input was illuminated was calculated, and PER was calculated based on both results. Bessel beam has been chosen because RP modes from centrosymmetric waveguides such as optical fiber have a Bessel shape solution [26]. In addition, RP light has zero intensity at the center generally due to circular symmetry. Thickness of the metal layer and the protective layer were adjusted to maximize the transmission. The period was assumed to be 100 nm, and duty ratio was selected to be 25%. Free-space wavelength was chosen as 1064 nm [27,28], which is commonly used in Erbium doped lasers.

Before calculating and optimizing the protected MSCRA structure, the transmission of MSCRA without the protective layer was optimized. The structure was optimized when the thickness of the metallic layer was 228 nm. The optimized transmittance was 79.82% when the light direction was from dielectric to air, and 79.86% vice versa.

**Figure 2:**
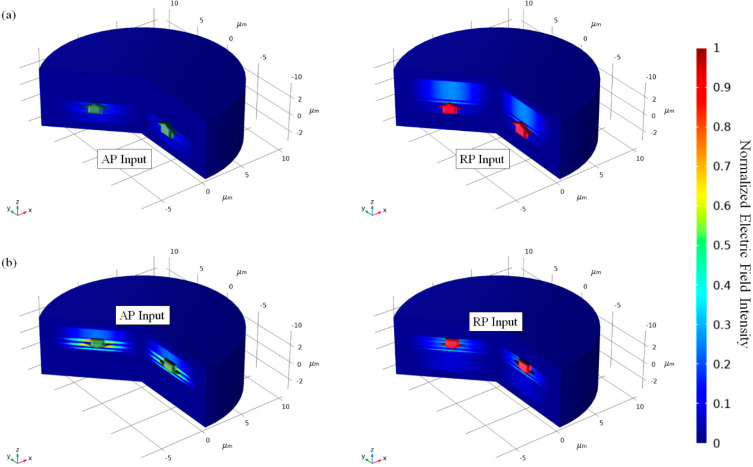
Electric field intensity pattern of protected MSCRA structure for each AP and RP inputs with direction of (**a**) Substrate to free-space and (**b**) Free-space to substrate, respectively.

The structure was optimized under condition at metal layer thickness of 160 nm and protective layer thickness of 375 nm. For the case from substrate to free-space, the calculated transmittance was 97.45% and the PER was 20.03 dB. For the case from free-space to substrate, the calculated transmittance was 97.51% and the PER was 19.96 dB. Figure 2 shows the electric field intensity pattern of both directions when RP and AP Bessel beam input was illuminated, respectively. As shown in Figure 2, it is shown that most of the RP components are transmitted but most of the AP components are reflected. It is worth noting the transmittance enhancement after the protective layer is ~22.1%.

### 3.2. MSCRA Based Dual-Focusing RP Lens

Once protected MSCRA is optimized, the structure can be applied to most binary concentric optical components. By simply replacing the opened region with protected MSCRA, a device has an additional function which transmits only the RP component while maintaining the characteristics of the original device. It can be applied to various optical devices such as focusing, multiple focusing, optical needle, and superoscillatory focusing. In this paper, an on-axis dual-focusing lens based on the virtual point method was designed and test via numerical simulations.

**Figure 3:**
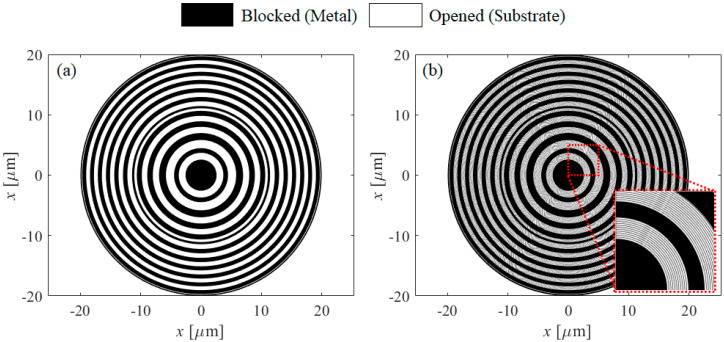
(**a**) The designed dual-focusing binary lens. (**b**) MSCRA assisted dual-focusing lens. The inset shows the magnified image.

Figure 3a shows the designed dual-focusing lens. The focal position was selected to be 10 μm and 15 μm. The diameter of the lens was selected to be 40 μm. The MSCRA assisted dual-focusing lens is shown in Figure 3b. The opened regions are filled with MSCRA structure. Inset shows the magnified view.

**Figure 4:**
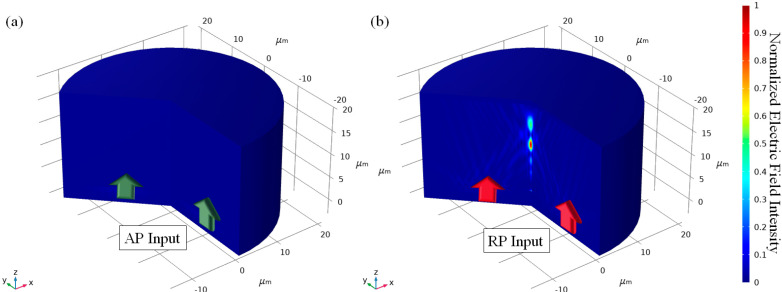
Normalized electric field pattern after protected MSCRA applied dual-focusing lens when the incident light is (**a**) Azimuthally polarized and (**b**) Radially polarized, respectively.

After designing the lens, a full vectorial numerical simulation has been performed to test the protected MSCRA assisted lens. The optimized value obtained on Section 3.1 has been used. Both AP and RP component Bessel beam has been illuminated, and the results are shown in Figure 4. Most of the AP component light has been prohibited to transmit the lens, but the RP component light has been focused on desired position, *z* = 10 μm and 15 μm.

## 4. Discussion

### 4.1. Protective Layer Thickness Dependency

In order to further analyze the phenomenon of the thickness of each layer, numerical simulations were performed by changing the thickness of the metal layer and the thickness of the protective layer. Both transmittance and PER were calculated, and properties were compared for both substrate to free-space and free-space to substrate directions.

**Figure 5:**
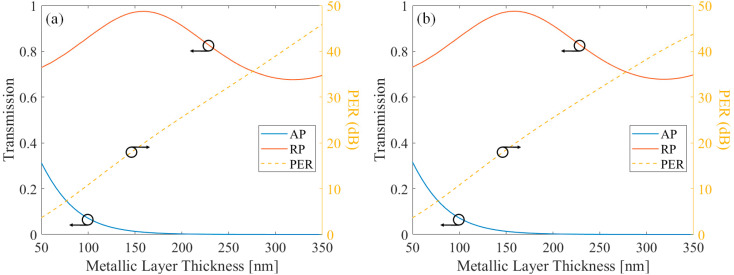
The AP component transmission, RP component transmission, and their PER of the protective layer applied MSCRA in terms of metallic layer thickness. The direction of the light is (**a**) Substrate to free-space and (**b**) Free-space to substrate.

First, simulations were performed by changing the metal layer thickness. Other properties were fixed at the optimized value. Figure 5 shows the transmission of AP and RP components, and the PER showing their ratio, in terms of the metal thickness. Figure 5a shows the case when light propagates from free-space to the substrate, and Figure 5b shows the case when light propagates from the substrate to the metal. First, it is shown that two different directions showed similar characteristics. The transmission was exponentially decayed according to the thickness of the metal in the case of the AP component input, and the oscillating phenomenon similar to the sine wave was observed in the case of the RP component input. This is because, based on the effective medium theory, the effective MSCRA medium has a metal-like property with a high imaginary part of the refractive index for the AP component, and a dielectric-like property with a low imaginary part of the refractive index for the RP component [19,29].

**Figure 6:**
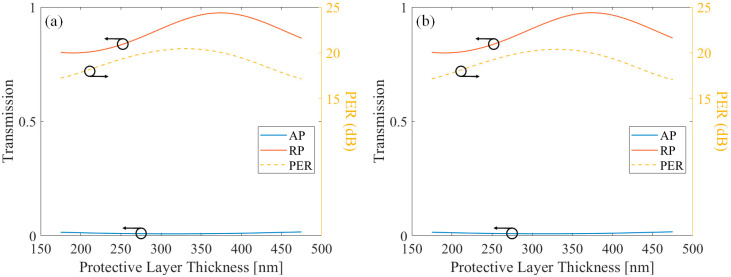
The AP component transmission, RP component transmission, and their PER of the protective layer applied MSCRA in terms of protective layer thickness. The direction of the light is (**a**) Substrate to free-space and (**b**) Free-space to substrate.

Subsequently, similar simulations were performed by changing the thickness of the protective layer. Other properties were fixed at the optimized value. Figure 6 shows the transmission of AP and RP components, and the PER showing their ratio, in terms of the protective layer thickness. Figure 6a shows the case when light propagates from the free-space to the substrate, and Figure 6b shows the case when light propagates from the substrate to the metal. Again, it is shown that two different directions showed similar characteristics.

According to the thickness of the protective layer, the transmission showed an oscillating result for both polarizations, as the protective layer is dielectric. As the metal thickness was enough to block the AP component, PER was dependent on the RP transmission [19,29].

By these results, it is shown that the MSCRA layer is a metal-like material for AP inputs and dielectric-like material for RP input. It is also shown that an additional design of freedom was available by controlling the thickness of the protective layer, and could optimize the efficiency.

### 4.2. Material Selection

In the previous discussion, the protective layer was selected as SiO_2_. The protective layer added MSCRA system can be considered a 4-layer system from the view of effective medium theory. In a 3-layer system, or single coating anti-reflection, the transmission is optimized when the refractive index of the medium layer is $\sqrt{n_{a}n_{s}}$, where *n_a_* and *n_s_* are the refractive index of air and substrate, respectively [15]. For a 4-layer system, or double-layer coating anti-reflection, the transmission is optimized when the refractive indices are $\sqrt[{3}]{n_{a}^{2}n_{s}}$ and $\sqrt[{3}]{n_{a}n_{s}^{2}}$, respectively [16,30]. For the case of silicon, the optimized refractive indices are 1.53 and 2.33, respectively. Therefore, the refractive index of the protectional material should be close to 1.53, and the effective refractive index of the metal-protective material mixed area should be close to 2.33. The effective index of the mixed area is engineerable by tuning the duty ratio of the metal, so the protective layer must be chosen with a refractive index around 1.53. These results show that SiO_2_ is a proper material for protection.

**Figure 7:**
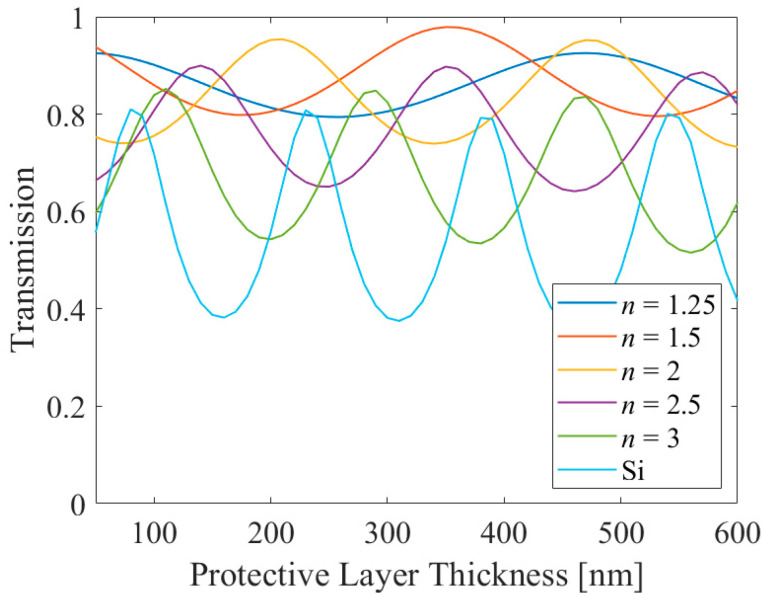
The component transmission of the protective layer applied MSCRA for various materials in terms of protective layer thickness.

First, simulation was performed by varying the refractive index and the thickness of the protectional material while fixing the other parameters. Note that as it is shown in the previous results, the results of substrate to air and air to substrate were almost similar, so only the situation of substrate to air is considered in this section. Figure 7 shows the result, and as expected a refractive index of 1.5 showed the highest transmission. Then, optimization was performed by varying the duty ratio and metal thickness, and the results are shown in Table 1. It is shown that the transmission is higher where the protective layer refractive index is near to the ideal value, 1.53. In addition, in terms of PER, higher metal ratio, i.e., high duty ratio and thick metal resulted in higher PER, where the metal absorbs the AP component. Also, it is shown that higher refractive index shows lower PER for similar metal thickness and duty ratio. This can be understood by effective medium theory, or also by intuition that the wavelength becomes short as the refractive index increases, so it may pass the metallic slit without difficulty.

### 4.3. Surface Structure

Previously, it was assumed that the surface of the structure was flat. However, in practice, if the MSCRA structure is fabricated first and then the protective layer is deposited, the thickness is generally the same at all regions [14,31,32]. That is, the surface also has a corrugated surface such as in a ring shape. Therefore, flat surface structure requires an additional process such as polishing.

The corrugated surface protective layer MSCRA, without polishing, is similar to a 5-layer system, and the additional dielectric-air grating layer shows lower effective refractive index compared to the protective layer, which will still act as a antireflection schematic. Additional simulations were performed in this corrugated surface case. As it is shown in the previous results, the results of substrate to air and air to substrate were almost similar, so only the situation of substrate to air is considered in this section.

**Figure 8:**
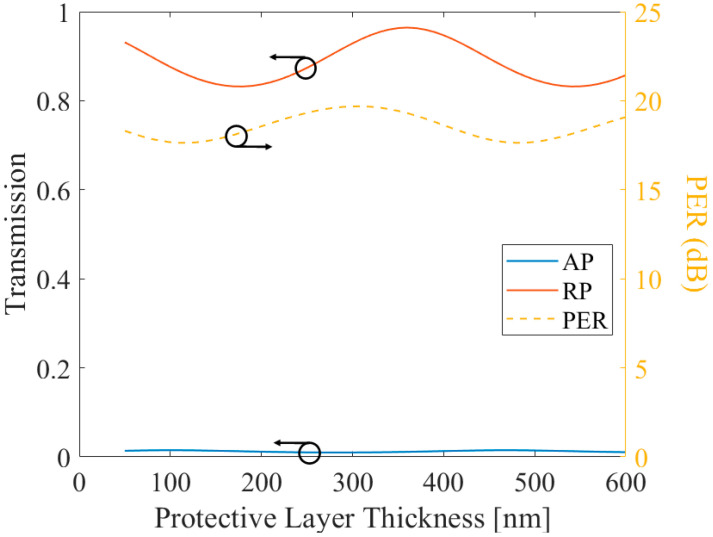
The AP component transmission, RP component transmission, and their PER of the corrugated surface protective layer applied MSCRA in terms of protective layer thickness.

Figure 8 shows that even when the surface is not polished, the structure shows high transmission and PER, which is optimized as 96.42% of transmission with 19.3 dB of PER when the metal thickness was 360 nm, and all other parameters are the same to optimized flat surface MSCRA case. Therefore, the protected MSCRA structure is also valid with corrugated surface, which has relatively low fabrication difficulty compared to flat surface structure.

## 5. Conclusions

To conclude, a method to increase the stability of the MSCRA for RP light selection has been proposed. Simply by attaching a dielectric layer above the MSCRA, the stability increases, and an additional design freedom is added by tuning the thickness of the protective layer. Numerical simulations have confirmed the idea and optimized the transmission, resulting in ~97.4% of RP component transmission with ~20 dB of PER.

In addition, application potential of protected MSCRA to a concentric, binary optical plate was also shown. The protected MSCRA has been applied on dual-focusing zone plate and showed similar dual-focusing characteristics while only transmitting the RP component.

The protective layer applied MSCRA schematic is believed to be applied on various optical zone plates, and could improve various applications such as depth sensing, bio-sensing, or optical machining.

## Figures and Tables

**Table 1:** Optimized results of the protected MSCRA structure of various materials.

Refractive Index	Duty Ratio	Metal Thickness	Protective Layer Thickness	RP Transmission	PER
1.25	0.3	180	435	93.45	26.81
1.5	0.25	145	375	97.96	17.39
2	0.25	225	415	97.13	25.71
2.5	0.25	190	120	91.41	18.87
3	0.3	170	100	86.63	17.40
3.55 (Si)	0.25	155	85	81.73	9.39

## Data Availability

Data are available within the article.
